# Improved Breakdown Strength of Polypropylene Film by Polycyclic Compounds Addition for Power Capacitors

**DOI:** 10.3390/ma14051185

**Published:** 2021-03-03

**Authors:** Ranran Xu, Jiwen Xing, Boxue Du, Meng Xiao, Jin Li

**Affiliations:** Key Laboratory of Smart Grid of Education Ministry, School of Electrical and Information Engineering, Tianjin University, Tianjin 300072, China; xuranran0512@126.com (R.X.); xingjiwen@163.com (J.X.); duboxue@tju.edu.cn (B.D.); lijin@tju.edu.cn (J.L.)

**Keywords:** flexible HVDC transmission, power capacitor, polypropylene film, conductivity, breakdown strength, polycyclic compounds

## Abstract

In this paper, an improved method for the electric performance of polypropylene (PP) film was proposed to promote the safety and stability of power capacitors. Modified PP films containing three different polycyclic compounds were prepared, which showed good thermal properties and decreased DC conductivity. The DC breakdown strength of the modified PP films under both positive and negative voltage is increased compared with that of the original film. The deep traps introduced by polycyclic compounds and the decreased carrier mobility give an explanation of the decreased DC conductivity. A quantum chemistry calculation was further performed to clarify the mechanism for improving electrical performance, presenting that polycyclic compounds with a high electron affinity and low ionization energy can capture high-energy electrons, protecting the PP molecular chain from attack, and then increase the breakdown strength. It is concluded that the modified PP films by polycyclic compounds have great potential in improving the insulating properties of power capacitors.

## 1. Introduction

With the advantages of failure-free commutation and good controllability, flexible high-voltage direct-current (HVDC) transmission has developed dramatically [1]. The DC converter valve is the core equipment of flexible HVDC systems, in which the power capacitors play a crucial role in suppressing voltage fluctuation and protecting the Insulated Gate Bipolar Transistor (IGBT) from damage [2]. Insulation failure of power capacitors will lead to serious converter valve faults, threatening the safe operation of the power system [3,4]. In addition, with the increasing voltage level, the insulation performance of power capacitors is more stringently required [5].

Power capacitors are composed of a polypropylene (PP) film and metalized electrode. As an insulation material, PP film has a dominant influence on the insulation properties of power capacitors [6,7]. In the long-term operation process, the breakdown of PP film affected by the electrothermal field leads to a decrease in capacity and lifetime, which is an important cause for the failure or even explosion of power capacitors [8,9]. The molecular chains of PP break and become small molecular products, leading to the formation of low-density regions, where collision ionization occurs and eventually leads to the breakdown of PP film [10]. Increased carrier concentration and carrier mobility due to a temperature rise in the capacitor increase dielectric loss and further aggravate the breakdown of PP film [11,12,13]. Therefore, it is of great significance to improve the breakdown field strength of PP film without increasing the dielectric loss for the safety and reliability of power capacitors.

Polycyclic compounds with the ability to capture high-energy electrons and modulate charge transport have been widely studied to improve the insulation properties of polymers [14,15]. Polycyclic compounds can overcome the disadvantage of the agglomeration of inorganic nanoparticles and have received extensive concern in the modification of polymer materials [16]. The advantage of polycyclic compounds without agglomeration is important for the application of PP film in capacitors, which can avoid the breakdown of the weak points of PP film. Polycyclic compounds with the ability to capture high-energy electrons can reduce electron energy and protect the molecular chain from an attack [15]. Previous studies mainly focused on the effect of polycyclic compounds on space charges or electric trees in polymers. Research shows that polycyclic compounds can suppress space charges or electric trees in polymers [17]. Polycyclic compounds may provide an approach to modify the breakdown strength of polymers [18]. However, there is still a lack of research on the effect mechanism of polycyclic compounds on the electric properties of PP film, such as conductivity and electrical breakdown strength.

This paper investigated the breakdown strength and conductivity of PP film modified by polycyclic compounds. The thermal properties, DC conductivity, and breakdown strength of PP films modified by three different polycyclic compounds were tested. Trap level distribution and carrier mobility were obtained by the method of surface potential decay (SPD). A quantum chemical calculation was employed to analyze the effect mechanism of polycyclic compounds on the insulation properties of the PP film. The breakdown strength is improved, and the conductivity is decreased by the addition of polycyclic compounds, showing that polycyclic compounds have a great potential in the modification of PP film employed in power capacitors.

## 2. Materials and Methods

**Figure 1:**
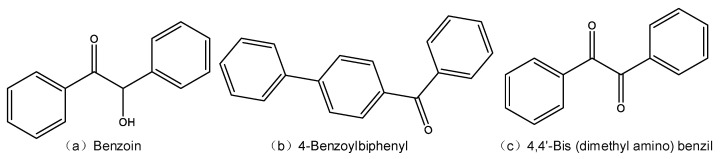
Molecular structures of the three kinds of polycyclic compounds.

The type of the PP resin used is PPH-T03. Three polycyclic compounds, benzoin, 4-benzoylbiphenyl, and 4,4′-bis (dimethyl amino) benzyl, were purchased from J&K Scientific Ltd., denoted by PC1, PC2, and PC3, respectively. The molecular structures of the three kinds of polycyclic compounds are shown in Figure 1. The polycyclic compounds were dispersed into the PP matrix by a Banbury mixer, and the filler content was 0.05 wt %. The mixing temperature was set at 190 °C, and the mixing process was continued for 20 min for homogeneous mixing. The PP resin and composite materials were made into 30 μm thick films by hot pressing. The filming process was continued for 30 min, and the pressure was set at 25 MPa. The PP films modified by PC1, PC2, and PC3 are denoted by PP-PC1, PP-PC2, and PP-PC3, respectively. Before the experiments, all the samples were dried and cleaned. For a clearer description, Table 1 gives the designation and composition of each type of sample.

The thermal properties of the samples were tested by differential scanning calorimetry (DSC, NETZSCH DSC200F3). DSC experiments of the samples were conducted from 50 to 180 °C, with the heating and cooling rate set at 10 °C per minute.

DC conductivity was measured by a three-electrode system, in which a cylindrical electrode with a diameter of 30 mm is used as the high-voltage anode, an electrode with a diameter of 25 mm is used as the cathode, and a ring electrode connected to the ground is used as the guard electrode. The measurement system of DC conductivity was placed in a temperature-controlled oven. The current of each sample was measured by an electrometer (Keithley 6517B) for 30 min. The mean value of the last 100 points of currents was taken as the steady-state conduction current. The DC conductivity was then calculated by the conduction current. The conductivity of the samples was measured from 25 to 85 °C. DC conductivity at different conditions was tested 15 times for precise measurement.

DC breakdown strength experiments were performed using a pair of copper ball-plate electrodes immersed in pure insulating oil [19]. A ramping DC voltage source with a speed of 500 V/s was used to conduct the breakdown experiment, and each kind of sample was tested 15 times for precise measurement. The breakdown experiments were conducted at 85 °C. The Weibull distribution was used to analyze the breakdown strength of the samples, and the value of the DC breakdown strength at a 63.2% probability was obtained.

In order to analyze the effect mechanism of polycyclic compounds on the conductivity and breakdown strength, quantum chemical methods were conducted. The density of states (DOS), energy level distribution, molecular orbitals (MOs), and 3D electrostatic potential distribution were calculated by quantum chemical methods based on density functional theory (DFT) with the B3LYP Hamiltonian and the 6–31 G basis function. MOs are constructed by atomic orbitals and hybrid orbitals of all atoms in a molecule. The self-consistent (SCF) method can be used to calculate the distribution of MOs [20]. The B3LYP hybrid functional method and the 6–31 G (d) basis were employed to study the DOS, energy levels, and 3D electrostatic potential of the molecules [21].

## 3. Results

### 3.1. Sample Characteristics

**Figure 2:**
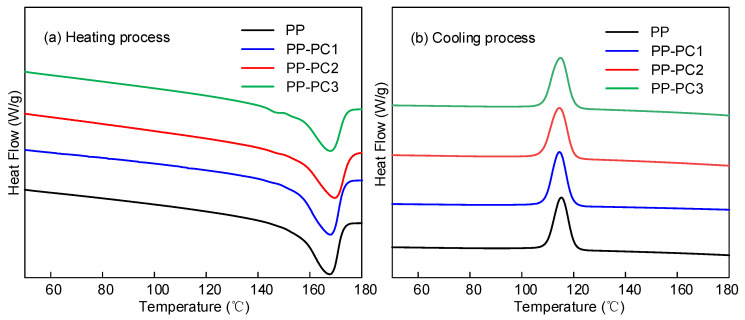
DSC curves of the samples: (**a**) heating process, (**b**) cooling process.

The thermal characteristics of PP film are important, for the temperature rise of power capacitors during operation is inevitable. The DSC spectrum was tested to study the thermal characteristics of PP film modified by polycyclic compounds, as shown in Figure 2.

The DSC of the samples was conducted from 50 to 180 °C, with the heating and cooling rate set at 10 °C per minute. The melting temperature can be obtained from the heating process shown in Figure 2a, and the crystallization temperature can be obtained from the cooling process shown in Figure 2b. The melting temperature (Tm), crystallization temperature (Tc), and enthalpy of the absorption during melting (Hm) can be obtained from Figure 2 and are presented in Table 2. The melting temperature peaks of PP-neat, PP-PC1, PP-PC2, and PP-PC3 are 167.48, 167.62, 169.60, and 167.56 °C, respectively. The addition of polycyclic compounds slightly increases the melting temperature peaks, which has little effect on the high temperature properties of PP films. The crystallization temperature peaks of PP-neat, PP-PC1, PP-PC2, and PP-PC3 are 115.31, 114.53, 114.60, and 114.91 °C, respectively. The crystallization temperature peaks of all the samples are almost the same. Crystallinity was calculated as the ratio of the absorbed enthalpy during the melting process to the enthalpy at 100% crystallinity. The enthalpy of PP at 100% crystallinity is 209 J/g. The crystallinity of PP-PC3 increased slightly, whereas the crystallinity of PP-PC1 and PP-PC2 decreased slightly. The crystallinity of all samples is above 30% with little difference. Compared with PP-PC1 and PP-PC2, PP-PC3 presents a slightly improved thermal property.

The conduction mechanism can be explained by hopping conduction. The process that charge carriers transport from one localized state to another localized state is referred to as “hopping transport”. The conduction current is related to the temperature, electric field, carrier density, and carrier mobility. The carrier mobility is related to the hopping distance and energy of carriers. The current density *J* is given as follow:

(1) $$J(E,T)=2ne\lambda \upsilon \exp(-\frac{eW}{k_{B}T})\sinh(\frac{\lambda eE}{2k_{B}T})$$

where *n* is the carrier density, *e* the electric charge of the carriers, *λ* the hopping distance, *ν* the frequency of thermal vibration, *W* is the activation energy, *k_B_* is the Boltzmann’s constant, *T* the temperature, and *E* is the electric field.

**Figure 3:**
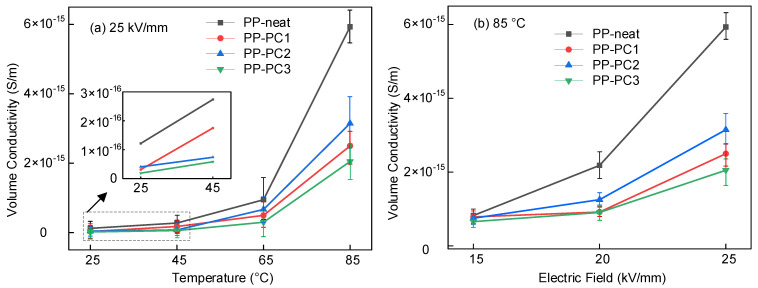
Relationship between the DC conductivity, electric field, and temperature (**a**) under 25 kV/mm (**b**) at 85 °C.

The DC conduction current was measured from 25 to 85 °C, and the value of DC conductivity was calculated based on the DC conduction current. Figure 3 shows the relationship between the DC conductivity, electric field, and temperature. In Figure 3a, the DC conductivity is greatly affected by temperature and increases with the increasing temperature. The DC conductivity is relatively lower at temperatures from 25 to 45 °C. When the temperature increases from 45 to 85 °C, the DC conductivity increases significantly. The charge carriers can obtain more kinetic energy at a higher temperature, and the transport process of carriers can be promoted. In Figure 3b, the conductivity increases with the increasing electric field because the higher electric field can enhance the directed migration of carriers. In Figure 3a,b, the conductivities of PP-PC1, PP-PC2, and PP-PC3 are lower than those of PP-neat, which indicates that the polycyclic compounds can suppress conduction loss. The DC conductivity of the sample modified by PC3 is smaller than other samples. The mechanism will be discussed in Section 4.

As shown in Figure 3, the DC conductivity increases at a high temperature and high electric field, which means more conduction loss. The increased conduction loss will lead to a higher temperature rise in the power capacitor. The vicious cycle between conduction loss and temperature rise speeds up the breakdown of PP film. Therefore, decreasing the DC conductivity is beneficial for the operation of power capacitors.

### 3.2. DC Breakdown Strength

**Figure 4:**
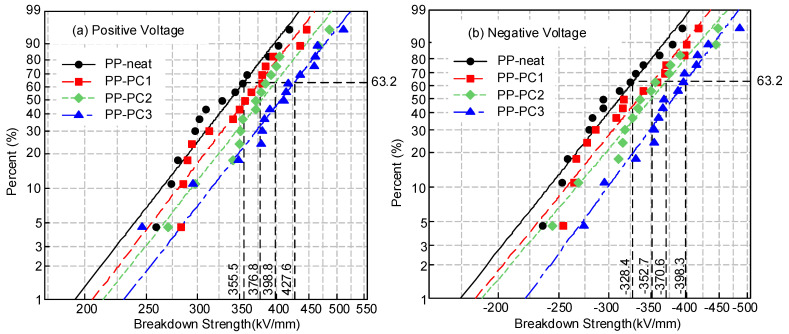
Weibull distribution of the DC breakdown strength of samples at 85 °C: (**a**) positive voltage; (**b**) negative voltage.

The DC breakdown strength of the samples was tested at 85 °C, and the data were analyzed using the Weibull distribution, as shown in Figure 4. The characteristic breakdown strength is defined as the breakdown field strength when the breakdown probability is 63.2%. When under positive voltage, as shown in Figure 4a, the characteristic breakdown strength values of PP-neat, PP-PC1, PP-PC2, and PP-PC3 are 355.5, 376.8, 398.8, and 427.6 kV/mm, respectively. When under negative voltage, as shown in Figure 4b, the characteristic breakdown strength values of PP-neat, PP-PC1, PP-PC2, and PP-PC3 are −328.4, −352.7, −370.6, and −398.3 kV/mm, respectively. The characteristic breakdown strength of films under both positive and negative voltages is improved by the addition of polycyclic compounds. It can be seen that the breakdown strength of PP-PC3 is highest. Based on the breakdown theory of high-energy electrons, the molecules of polymers attacked by high-energy electrons will break and form low-density domains. The breakdown of polymers will then initiate from these low-density domains. Therefore, the protection of PP molecular chains from the attack of high-energy electrons is of great importance for improving breakdown strength. Polycyclic compounds can protect PP molecular chains from the attack of high-energy electrons, which improves the breakdown strength. The effect mechanism of polycyclic compounds on breakdown strength will be discussed in Section 4.

**Figure 5:**
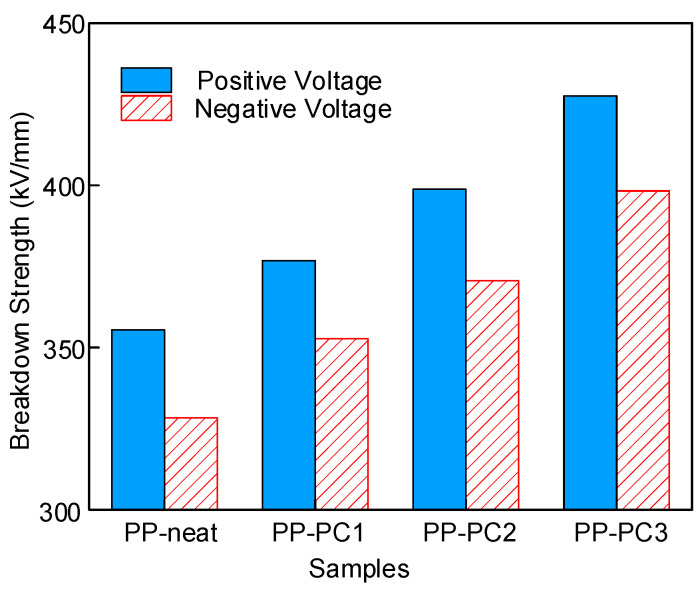
Characteristic breakdown strength of samples at 85 °C.

The absolute values of characteristic breakdown strength under positive and negative voltages are shown in Figure 5. The breakdown strength is higher under positive voltage than that under negative voltage. The injected number of electrons is larger than the number of holes based on the theory of charge injection and extraction [22]. A large number of electrons will be injected from the high-voltage electrode when under negative voltage. The electrons obtain energy and become high-energy electrons under a high electric field and high temperature, which attack and break the molecular chains. The broken molecular chains form a low-density domain, where the breakdown occurs. Therefore, the breakdown strength of samples is lower under negative voltage.

## 4. Discussion

### 4.1. Effect of Deep Traps on DC Conductivity

**Figure 6:**
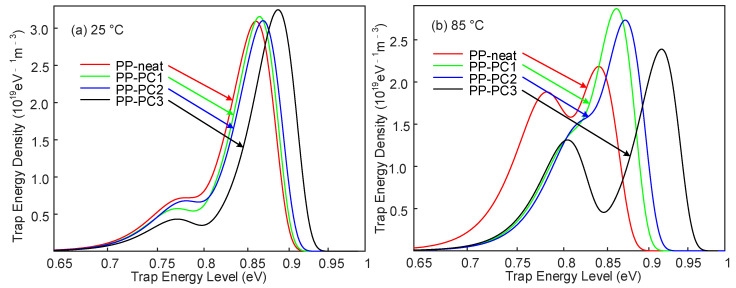
Trap level distribution of samples at (**a**) 25 and (**b**) 85 °C.

The trap level distribution directly affects the carrier transport process, which is closely related to the DC conductivity and DC breakdown strength. In order to understand the effects of polycyclic compounds on DC conductivity and DC breakdown strength, the trap energy distribution needs to be analyzed. The trap level distribution of the samples was obtained by the method of SPD [23]. Since the trap level distribution at different temperatures has a similar trend, Figure 6 only shows the trap level distributions at 25 and 85 °C. The trap energy density presents two peaks. The lower energy level corresponding to the energy density peak is defined as the shallow trap level (STL), and the higher energy level corresponding to the energy density peak is defined as the deep trap level (DTL). Charge carriers can easily trap and detrap from the STLs; therefore, the STLs will promote the conduction process. It is hard for charge carriers to detrap from the DTLs, which will suppress the transport of carriers. The trapped carriers in DTLs can form an electric field opposite to the applied electric field, which can inhibit the injection of carriers from the electrode.

In Figure 6a, the trap energy density (TED) of STL in PP film is highest at 25 °C. The STL of PP-PC1, PP-PC2, and PP-PC3 are deeper than the STL of PP-neat, and the TEDs of STL of PP-PC1, PP-PC2, and PP-PC3 are lower than that of PP-neat. It also can be obtained that the DTL of PP-PC1, PP-PC2, and PP-PC3 are deeper than the DTL of PP-neat. PP-PC3 presents the deepest DTL, and the TED of DTL of PP-PC3 is the highest as well. The trap energy distribution at 85 °C, shown in Figure 6b, presents a similar trend with the trap energy distribution at 25 °C shown in Figure 6a. Both the STL and DTL of PP films modified by polycyclic compounds are deeper than that of PP-neat at 85 °C. Furthermore, the PP-PC3 presents the deepest DTL at 85 °C. Based on the results of the trap level distribution shown in Figure 6, it can be inferred that the polycyclic compounds can introduce DTLs. The introduced DTLs by polycyclic compounds can suppress the transport of carriers, which will result in a decreased DC conductivity.

The hopping conduction theory holds that the carrier hopping transport in a localized state is the main process of the conduction current. The expression of the current density also indicates that the conduction current is closely related to the charge carrier mobility. The carrier mobility reflects the average transport rate of charge carriers.

**Figure 7:**
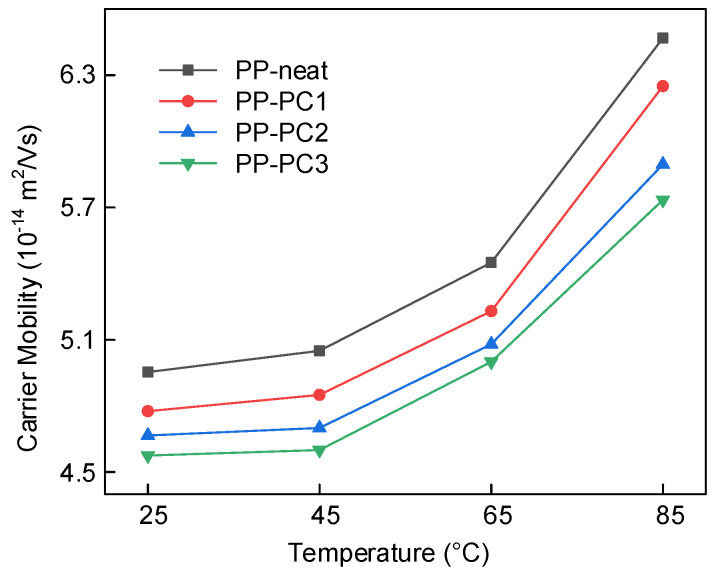
Relationship between the carrier mobility and the sample temperature.

The carrier mobility at different temperatures was obtained by the method of SPD [24], as shown in Figure 7. It can be obtained that the temperature greatly affects the carrier mobility, and the carrier mobility increases at high temperatures. More energy can be obtained by charge carriers at higher temperatures, which makes charge carriers detrap much easier. The increased detrapping probability leads to increased transport of the charge carrier from one trap to another trap, which increases the carrier mobility. Since there is a positive correlation between conductivity and carrier mobility, the trend of carrier mobility with temperature explains the rise of conductivity with temperature, as shown in Figure 3.

It also can be obtained that the carrier mobility of PP-PC1, PP-PC2, and PP-PC3 is lower than that of PP-neat. The decreased carrier mobility can be explained by the introduced DTLs by polycyclic compounds. It is hard for trapped charges to detrap from DTLs because the trapped charges need more energy to detrap from the DTLs. The PP-PC3 presents the lowest carrier mobility, which is related to the deepest DTLs of PP-PC3. The decreased carrier mobility leads to the lower DC conductivity of PP-PC1, PP-PC2, and PP-PC3, as shown in Figure 3. The lowest carrier mobility explains the mechanism of the lowest DC conductivity of PP-PC3 at different temperatures.

### 4.2. Effect of Polycyclic Compounds on Breakdown Properties

A quantum chemistry calculation was employed to explore the effect mechanism of polycyclic compounds. Firstly, the PP-neat and polycyclic compounds were modeled using Gaussian View. Gaussian 09 was then used to calculate the DOS, energy level distribution, MOs, and 3D electrostatic potential distribution [20].

**Figure 8:**
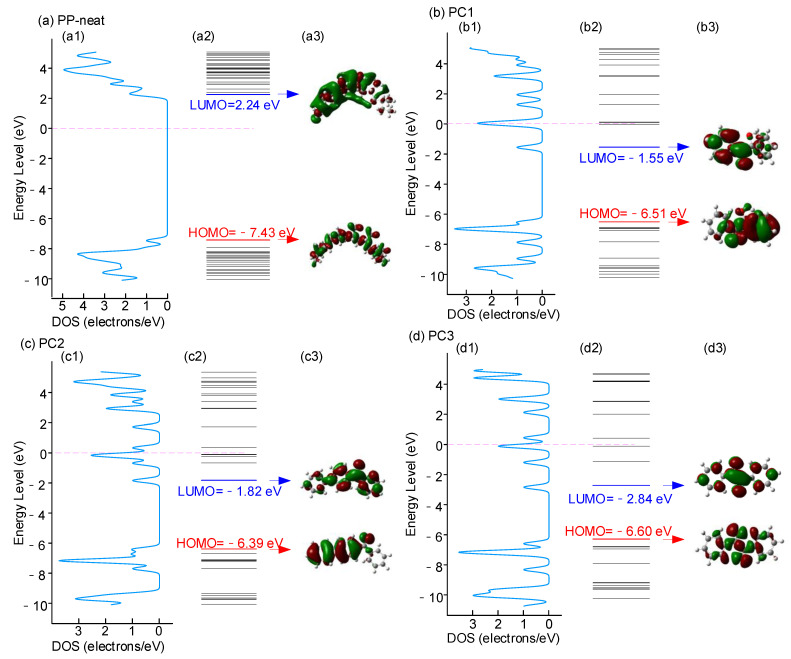
DOS, energy level distribution, and molecular orbitals (MOs) of the samples: (**a1**–**d1**) DOS, (**a2**–**d2**) energy level distribution, and (**a3**–**d3**) MOs.

The DOS, energy level distribution, and MOs of the PP-neat and polycyclic compounds are shown in Figure 8. Comparing the DOS of PP-neat, PC1, PC2, and PC3 in Figure 8(a1–d1), the polycyclic compounds have some electron traps and hole traps in the range of band gaps of PP-neat, which means polycyclic compounds can introduce electron traps and hole traps. Figure 8(a2–d2) presents the energy level distribution. The highest occupied molecular orbital (HOMO) and lowest unoccupied molecular orbital (LUMO) levels are marked with red and blue lines, respectively. For PP-neat, the LUMO is 2.24 eV, and the HOMO is −7.43 eV. The LUMOs of PC1, PC2, and PC3 are negative and much lower than the LUMO of PP-neat, and the HOMOs of all polycyclic compounds are higher than the HOMO of PP-neat. The energy level distribution also indicates that polycyclic compounds can introduce energy levels in PP. The DOS and energy level distribution verify the experimental results of SPD in Figure 6.

Figure 8(a3–d3) presents the MOs of LUMO and HOMO. The MOs present the wave function characteristics of an electron in the molecules, which is related to the probability of electron occurrence. For PP-neat in Figure 8(a3), the MO at LUMO locates at the surface of the molecular chain, indicating that an injected electron will travel outside the backbone of the molecular chain. The injected electrons prefer to travel among the backbones, such as the free volumes among chains or voids, where they can easily accelerate and become high-energy electrons. The PP molecular chain will break when attacked by high-energy electrons.

The MO at the HOMO level distributes inside the backbone, indicating that the orbital electrons of PP-neat at the HOMO level travel inside the molecular chains, making it easy for PP-neat to break. The MOs at the LUMO and HOMO levels of polycyclic compounds distribute around the molecular chains, as shown in Figure 8(a3–d3). Benzene rings and carbonyl groups are the centers that attract additional electrons. The benzene ring structure forms a large π bond through SP2 hybridization, forming a chemical trap with a large charge capture cross-section and strong capture ability. The carbonyls in polycyclic compounds also act as a chemical trap.

The electron affinity (χ), ionization energy (φi), and band gap were calculated based on the energy level distribution, as shown in Table 3. The electron affinity χ is the energy difference between the vacuum level and the LUMO level. The electron affinity χ of PP-neat is −2.24 eV, whereas the electron affinities of PC1, PC2, and PC3 are 1.55, 1.82, and 2.84 eV. As a result, the polycyclic compounds with positive χ are easier to attract electrons than PP-neat. Polycyclic compounds can absorb and attenuate the energy of high-energy electrons, and the absorbed energy will be released by unharmful methods, such as lattice vibration. The high-energy electrons then become low-energy electrons, which cause little destruction to PP molecular chains.

This explains that the addition of polycyclic compounds can capture high-energy electrons and protect the polymer matrix under high electric fields. The protection of molecular chains is beneficial for the improvement of the electrical breakdown strength of PP film. The carbonyl group and benzene ring increase the electron affinity χ of polycyclic compounds, as discussed in the preceding paragraph. The contribution of the carbonyl group is greater than that of the benzene ring in increasing the capturing ability of electrons; that is to say, the electron affinity. PC3 has the highest electron affinity compared with PC1 and PC2 because PC3 has the most carbonyl groups. The higher the electron affinity, the more protection for PP molecular chains. The ionization energy (φi) of PP-neat is 7.43 eV, whereas the electron affinities of polycyclic compounds are 6.51, 6.39, and 6.6 eV. As a result, the polycyclic compounds with lower φi are easier to attract hole charges than PP-neat. The band gaps of PP-neat, PC1, PC2, and PC3 are 9.67, 4.96, 4.57, and 3.76 eV, respectively. The decreased band gap indicates that the addition of polycyclic compounds will introduce some trap energy levels to PP film.

**Figure 9:**
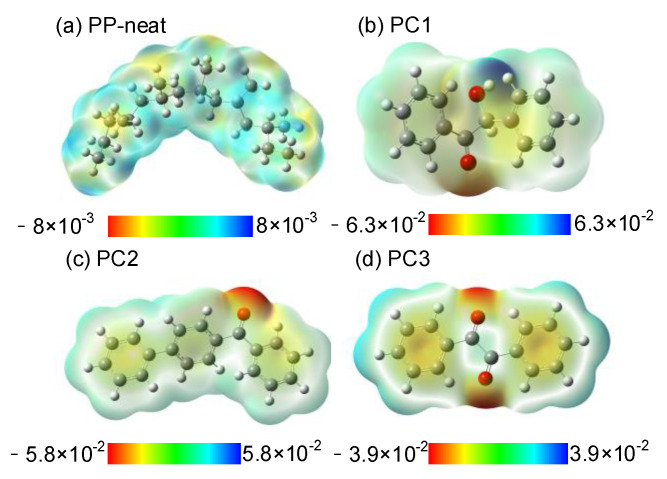
Three-dimensional (3D) electrostatic potential of samples, (**a**) PP-neat, (**b**) PC1, (**c**) PC2, and (**d**) PC3.

The 3D electrostatic potential distribution of PP-neat and polycyclic compounds was calculated, as shown in Figure 9. In Figure 9, the warm colors represent negatively charged, and the cold colors represent positively charged, respectively. The number unit of the gradient color is eV in Figure 9. The orbital electron deviates in molecular chains for the difference in electronegativity between the carbon and hydrogen in PP-neat. For the polycyclic compounds, both the warm colors and cold colors were deepened, especially at the regions nearing benzene rings and carbonyl groups, meaning that both the electrons and holes can be captured much more easily.

The results of DOS, energy level, molecular orbitals (MOs), and 3D potential distribution verify the ability to capture high-energy electrons of polycyclic compounds and explain the mechanism of changing the energy level distribution.

## 5. Conclusions

In this paper, the effect mechanism of polycyclic compounds on the DC breakdown strength of PP film in a power capacitor was investigated. The improved DC breakdown strength with the addition of polycyclic compounds presents great potential for PP film modification, which is of great significance for the operation of power capacitors. The addition of polycyclic compounds slightly increases the melting temperature, which is beneficial for PP film operating at higher temperatures. PP-PC3 presents the best thermal property compared with PP-PC1 and PP-PC2. The DC breakdown strength of the PP film modified by polycyclic compounds at 85 °C is improved. The PP film modified by polycyclic compounds shows lower DC conductivity at different temperatures. The PP film modified by 4,4′-bis (dimethyl amino) benzyl presents the highest DC breakdown strength and lowest DC conductivity. The trap level distribution is affected by the addition of polycyclic compounds through the SPD measurement, which is closely related to the DC breakdown strength and DC conductivity. The addition of polycyclic compounds into the PP films introduces deep trap levels, and the trap level of the PP film modified by 4,4′-bis (dimethyl amino) benzyl is deepest. The DOS, energy level, MOs, and 3D potential distribution obtained by the quantum chemistry calculation analyze the effect mechanism of polycyclic compounds. The benzene rings and carbonyls of polycyclic compounds act as charge capture centers, which limits the carrier mobility and results in a decreased conductivity. The higher capability of capturing high-energy electrons of polycyclic compounds protects the molecular chains, which increases the DC breakdown strength of PP film.

The research results reveal that the selected polycyclic compounds have great potential for use in PP film modification for power capacitor applications. The PP films in this paper were made by hot pressing. Furthermore, PP film used in power capacitors is biaxially oriented polypropylene (BOPP) film. In future research, the effect of polycyclic compounds on BOPP film will be studied to analyze the influence of the bidirectional drawing process on polycyclic compounds.

## Figures and Tables

**Table 1:** Designation and composition.

Designation	Composition
PP-neat	PP film
PC1	Benzoin
PC2	4-benzoylbiphenyl
PC3	4,4′-Bis (dimethyl amino) benzyl
PP-PC1	PP film modified by benzoin
PP-PC2	PP film modified by 4-benzoylbiphenyl
PP-PC3	PP film modified by 4,4′-bis (dimethyl amino) benzyl

**Table 2:** Thermal parameters of samples.

Sample	Tm (°C)	Tc (°C)	Xc (%)
PP-neat	167.48	115.31	35.13
PP-PC1	167.62	114.53	35.32
PP-PC2	169.60	114.60	34.11
PP-PC3	167.56	114.91	36.19

**Table 3:** Energy level parameters of samples.

Energy Level Parameters	PP-Neat	PC1	PC2	PC3
Electron affinity χ (eV)	−2.24	1.55	1.82	2.84
Ionization energy φi (eV)	7.43	6.51	6.39	6.6
Band gap (eV)	9.67	4.96	4.57	3.76

## Data Availability

Data sharing is not applicable to this article.
